# Cangfu Daotan decoction with Diane-35 in phlegm-dampness polycystic ovary syndrome: a meta-analysis and systematic review

**DOI:** 10.3389/fmed.2026.1761111

**Published:** 2026-02-20

**Authors:** Xiaomeng Zhou, Yun Wang

**Affiliations:** Department of Obstetrics and Gynecology, Hangzhou Women’s Hospital (Hangzhou Maternity and Child Health Care Hospital), Hangzhou, Zhejiang, China

**Keywords:** Cangfu Daotan decoction, Diane-35, meta-analysis, phlegm-dampness, polycystic ovary syndrome

## Abstract

**Background:**

To systematically evaluate the efficacy of Cangfu Daotan decoction combined with Diane-35 in treating phlegm-dampness polycystic ovary syndrome (PCOS) and its effects on endocrine- and metabolism-related outcomes.

**Methods:**

Major English and Chinese databases (PubMed, Embase, Cochrane Library, Web of Science, CNKI, Wanfang, CQVIP, and SinoMed) were searched. Randomized controlled trials comparing Cangfu Daotan-based therapy plus Diane-35 versus Diane-35-based regimens were included. Two reviewers independently screened studies, extracted data, and assessed the risk of bias via the Cochrane tool. The meta-analysis was conducted with RevMan 5.4. The relative risk (RR) with 95% confidence intervals (CIs) was used for dichotomous outcomes, and the mean difference (MD) with 95% CIs was used for continuous outcomes. Fixed- or random-effects models were applied according to heterogeneity (*I*^2^ threshold of 50%). Publication bias was assessed via funnel plots.

**Results:**

Sixteen trials were included. Compared with the controls, the combination therapy significantly improved the total effective rate (RR = 1.24, 95% CI 1.18–1.31, *p* < 0.00001). It also significantly reduced serum testosterone (MD = −0.26, 95% CI –0.37 to −0.15, p < 0.00001), luteinizing hormone (MD = −1.64, 95% CI –2.46 to −0.82, *p* < 0.0001), follicle-stimulating hormone (MD = −0.62, 95% CI –1.11 to −0.13, *p* = 0.01), and the LH/FSH ratio (MD = −0.27, 95% CI –0.43 to −0.12, *p* = 0.0006). BMI also decreased (MD = −1.55, 95% CI –3.06 to −0.04, *p* = 0.04). Funnel plots suggested no obvious publication bias. Overall risk-of-bias assessment indicated insufficient reporting or implementation of blinding and unclear allocation concealment.

**Conclusion:**

Cangfu Daotan decoction combined with Diane-35 may enhance clinical efficacy and improve key hormonal indicators and BMI in patients with phlegm-dampness-related PCOS. However, the evidence is limited by methodological weaknesses and heterogeneity across studies; well-designed, large-scale RCTs are needed to confirm these findings.

## 1 Introduction

Polycystic ovary syndrome (PCOS) is one of the most common endocrine illnesses, affecting up to 10% of women of childbearing age (1). PCOS manifests clinically primarily through amenorrhea, and hyperandrogenaemia. Western medicine interventions for PCOS mainly involve medications such as clomiphene (2), Diane-35 (3), and metformin (4), which aim to reduce testosterone (T) levels, improve the menstrual cycle, and address insulin resistance. Although Traditional Chinese Medical (TCM) literature does not explicitly mention “PCOS,” relevant content can be found in chapters related to clinical manifestations such as “hypomenorrhea” and “amenorrhea” (5). TCM’s constitutional theory aids in understanding PCOS by categorizing it into different syndromes on the basis of clinical manifestations and biochemical characteristics. In TCM, PCOS-related presentations are categorized into several syndrome patterns. In this review, we focus specifically on the ‘phlegm-dampness’ pattern because it is frequently reported in clinical trials and is often discussed in relation to metabolic manifestations (6). The phlegm-dampness constitution PCOS, a TCM defined constitution, is prevalent in PCOS patients and closely linked to living conditions, eating habits and mental states (7). For readers unfamiliar with TCM, ‘phlegm-dampness’ is a pattern descriptor that commonly overlaps with a metabolic phenotype (e.g., higher BMI/central adiposity and related metabolic disturbances).

In addition to common PCOS symptoms, phlegm damp PCOS exhibits TCM characteristics such as a plump abdomen, chest tightness, phlegm, drowsiness, body weight discomfort, an appetite for fat and sweet food, a fat tongue, and white and greasy tongue coating. TCM drugs, which are selected on the basis of the patient’s syndrome, are often used alongside basic Western medicine to treat phlegm-dampness PCOS (8).

Cangfu Daotan decoction, which originated from the consilia of Ye Tianshi, a renowned TCM doctor, is a frequently employed remedy for phlegm-dampness PCOS. In contrast to Atractylodes, Fructus risaema, Pinellia risaem, Nutgrass galingale rhizome, Dried tangerine peel, Poria cocos, Bile risaema, Ginger juice, Liquorice, and Medicated leaven, it addresses mainly physical obesity, excessive phlegm and salivation, blood stagnation, and irregular menstruation in women.

The rationale for investigating the combined use of Cangfu Daotan decoction with Diane-35 lies in the complementary mechanisms of action between TCM and Western medicine. Diane-35 is known for its efficacy in regulating hormonal imbalances and the menstrual cycle, whereas Cangfu Daotan decoction targets the underlying phlegm-dampness constitution, which is often not addressed by Western treatments alone. Previous studies have focused mostly on investigations of PCOS. Phlegm-dampnessis associated with metabolic dysregulation and endocrine imbalance, which may contribute to hyperandrogenism, menstrual irregularity, and insulin resistance in individuals with PCOS. Therefore, investigating Cangfu Daotan decoction as an adjunct to standard therapy may help address both endocrine and metabolic manifestations. From a TCM perspective, the phlegm-dampness pattern is proposed to interfere with reproductive function. In biomedical terms, this may overlap with metabolic and endocrine dysregulation which can impair ovarian responsiveness and contribute to menstrual irregularity in PCOS patients. Therefore, this study mainly explores the application value of Cangfu Daotan decoction in phlegm-dampness type PCOS.

## 2 Methods

### 2.1 Search methods

A comprehensive literature search was conducted across major international and Chinese databases to identify all relevant studies evaluating Cangfu Daotan-based interventions combined with Diane-35 for phlegm-dampness polycystic ovary syndrome (PCOS). The following electronic databases were searched from their inception to May 2025: PubMed/MEDLINE, Embase, Web of Science, the Cochrane Central Register of Controlled Trials (CENTRAL), CNKI (China National Knowledge Infrastructure), WanFang Data, the VIP Database (CQVIP), and SinoMed.

The search strategy incorporated both Medical Subject Headings (MeSH) and free-text terms. The main keywords used included: “Cangfu Daotan decoction”, “Cangfu Daotan”, “Cang Fu Dao Tan”, “Diane-35”, “polycystic ovary syndrome”, “PCOS”, “phlegm-dampness syndrome”, “traditional Chinese medicine”, “randomized controlled trial”, and their Chinese equivalents. Boolean operators (“AND”, “OR”), truncation marks, and field tags were applied as appropriate, (Cangfu Daotan decoction OR Cangfu Daotan OR Cang Fu Dao Tan), (PCOS OR polycystic ovary syndrome), (phlegm-dampness syndrome OR traditional Chinese medicine) and (“Diane-35”).

In addition to database searches, the reference lists of eligible articles and relevant reviews were manually screened to identify additional studies. To minimize publication bias, we also searched clinical trial registries (e.g., https://ClinicalTrials.gov), conference abstracts, and gray literature sources. No gray literature was excluded from the review. We specifically included conference abstracts and unpublished studies that met the eligibility criteria. Any relevant gray literature was assessed for quality and included in the final analysis if it met the predefined inclusion criteria.

The search results were imported into EndNote for deduplication. Two reviewers independently screened titles and abstracts, followed by full-text assessment on the basis of predefined inclusion and exclusion criteria. Any disagreements were resolved through discussion or by consultation with a third reviewer.

### 2.2 Inclusion and exclusion criteria

#### 2.2.1 Inclusion criteria

Studies were considered eligible if they met all of the following criteria:

*Population*: Women were diagnosed with polycystic ovary syndrome (PCOS) according to recognized diagnostic standards (e.g., the Rotterdam criteria or equivalent) and classified as having phlegm-dampness syndrome on the basis of traditional Chinese medicine (TCM) pattern identification. No restrictions were imposed on age, ethnicity, or baseline hormonal levels. Definition of phlegm-dampness syndrome (TCM pattern identification): In this review, phlegm-dampness syndrome was defined using prespecified TCM pattern-identification criteria. Eligible participants were required to meet at least three major symptoms and/or two minor symptoms, together with characteristic tongue and pulse features.

*Major symptoms were defined as follows*: (1) overweight/obesity or central adiposity; (2) a sensation of heaviness of the body or limbs; (3) menstrual irregularity (oligomenorrhea/amenorrhea), often with scanty menstrual flow; (4) profuse, sticky (or greasy) leukorrhea; (5) chest/epigastric oppression, fullness or abdominal distension; (6) nausea or a tendency to retch, with excessive sputum/phlegm; (7) edema or a feeling of puffiness.

*Minor symptoms included (any 2 or more of the following)*: (1) poor appetite; (2) fatigue, somnolence, or lack of energy; (3) loose stools or diarrhea; (4) dizziness or a heavy-headed sensation; (5) palpitations or shortness of breath on exertion; and (6) sticky sensation in the mouth and/or thirst with little desire to drink.

*Tongue and pulse signs included*: a pale or light-red, swollen tongue (sometimes with teeth marks) with a thick, greasy white coating (or greasy coating), and a slippery (hua), soggy (ru), or soggy-slippery pulse.

These criteria were adapted from commonly used national TCM syndrome-differentiation standards and standard TCM gynecology textbooks (please replace with the exact guideline/consensus you followed in this review).

*Intervention*: Trials in which the treatment group received Cangfu Daotan Prescription, including the classical decoction, modified formulations, or Cangfu Daotan Pill, in combination with Diane-35 or other Diane-35-based regimens.

*Comparator*: Studies in which the control group received Diane-35 alone or Diane-35 combined with other standard Western medications (e.g., metformin), which is consistent with conventional management strategies for PCOS.

*Outcomes*: Studies reporting at least one predefined outcome were eligible. The primary outcomes were serum testosterone (T) and the LH/FSH ratio. The secondary outcomes included luteinizing hormone (LH), follicle-stimulating hormone (FSH), body mass index (BMI), total effective rate, and adverse events. For continuous outcomes (LH, FSH, T, LH/FSH, BMI), we extracted post-treatment values (or changes-from-baseline when consistently reported) with corresponding units. The total effective rate was extracted as reported in each trial and was generally defined as the proportion of participants classified as “recovered,” “markedly improved,” or “improved.”

*Study design*: Randomized controlled trials (RCTs) or controlled clinical trials directly comparing a Cangfu Daotan-based regimen with a Diane-35-based control regimen.

*Availability of data*: Studies providing extractable quantitative data for outcomes of interest, including baseline and post-treatment measurements or effect estimates.

*Diagnostic criteria for phlegm-dampness syndrome*: The diagnostic criteria for phlegm-dampness syndrome varied across the included studies. To ensure clarity, we have summarized the specific Traditional Chinese Medicine (TCM) standards and diagnostic criteria applied in each study in the table below. While the studies adhered to TCM pattern identification for phlegm-dampness syndrome, there were differences in the exact criteria used. These differences were carefully considered in our analysis. The major symptoms generally included overweight, abdominal fullness, nausea, and menstrual irregularity, with minor symptoms such as fatigue, dizziness, and sticky mouth. Table 1 detailing the diagnostic criteria used in each study is provided below for transparency.

**Table 1:** Diagnostic criteria for phlegm-dampness in included studies.

Study	TCM standard used	Diagnostic criteria for phlegm-dampness syndrome	References
Study 1	Chinese Medicine Clinical Guidelines	Major symptoms: Overweight, menstrual irregularity, sticky leukorrhea, nausea. Minor symptoms: Fatigue, dizziness, sticky mouth.	National Health Commission of the People’s Republic of China (2019) (20)
Study 2	Guidelines for TCM Diagnosis of PCOS	Major symptoms: Abdominal fullness, nausea, chest oppression. Minor symptoms: Palpitations, poor appetite.	National TCM Diagnostic Guidelines (2020) (21)
Study 3	Chinese Medicine Textbook for Gynecology	Major symptoms: Overweight, edema, profuse leukorrhea. Minor symptoms: Fatigue, heavy sensation in body.	Li. and Zhang, TCM Gynaecology Textbook (2018)
Study 4	National TCM Diagnostic Standards	Major symptoms: Obesity, menstrual irregularity, greasy tongue coating. Minor symptoms: Low energy, poor digestion.	National TCM Diagnostic Standards (2017) (22)

#### 2.2.2 Exclusion criteria

Studies were excluded if they met any of the following conditions:

Non-controlled or non-randomized designs, including observational studies, case series, case reports, reviews, conference abstracts, animal studies, or *in vitro* investigations.
Non-eligible populations, such as studies not involving PCOS patients, lacking standardized diagnostic criteria, or not specifying phlegm-dampness syndrome on the basis of TCM differentiation.
Non-eligible interventions, including studies not using Cangfu Daotan Prescription (original decoction, modified formulation, or pill) or those not combining the intervention with Diane-35.
Inappropriate comparators, such as studies in which the control group did not receive Diane-35-based regimens.
Insufficient data availability, including studies lacking extractable quantitative outcome data or incomplete reporting even after attempts to contact authors.
Duplicate publications or overlapping datasets, in which case the most complete and updated version of the study was included.
Studies with unclear treatment durations or major protocol deviations, preventing meaningful comparisons across trials.

### 2.3 Quality assessment and data extraction

Two reviewers independently assessed the methodological quality of the included studies and extracted the relevant data using standardized procedures.

1. *Risk of bias assessment*: The methodological quality of each study was evaluated using the Cochrane Risk of Bias Tool, as recommended in the Cochrane Handbook (version 5.3) (9). This tool examines seven domains: random sequence generation, allocation concealment, blinding of participants and personnel, blinding of outcome assessment, completeness of outcome data, selective reporting, and other potential sources of bias. Each domain was judged as having a low, unclear, or high risk of bias. Any discrepancies between the two reviewers were discussed and resolved through consultation with a third investigator.
2. *Study selection and data extraction*: Study screening, data extraction, and quality assessment were conducted independently by two researchers, followed by cross-checking to ensure consistency. NoteExpress was used to manage the literature, and Excel was used for structured data extraction. When essential data were incomplete or unclear, attempts were made to contact the original authors for clarification.
3. *Data extraction methods*: Data extraction was performed independently by two reviewers using a standardized data extraction form. Relevant information was extracted from each study, including study design, participant characteristics, interventions, outcomes, and results. Discrepancies in data extraction were resolved through discussion or by consulting a third reviewer. The data extraction process was double-checked to ensure accuracy, with a focus on ensuring the consistency of outcome measures (e.g., serum testosterone levels, LH/FSH ratio, BMI). Information on the risk of bias and other quality assessments was also recorded during the extraction process.
4. *Baseline comparability across studies*: To assess baseline comparability across studies, we systematically compared baseline characteristics (e.g., age, BMI, hormonal levels, and clinical symptoms) of participants between studies. We ensured that studies with significantly different baseline characteristics were addressed appropriately in the analysis, and sensitivity analyses were conducted to explore the impact of these differences. If significant baseline imbalances existed between the intervention and control groups in individual studies, we noted them as potential sources of heterogeneity. Where applicable, we reported any measures taken to adjust for baseline differences, such as statistical adjustments in the analysis (e.g., controlling for baseline BMI in regression models).

### 2.4 Data collection and analysis

RevMan 5.4 software was employed for data analysis. The relative risk (RR) with a 95% confidence interval (CI) was used as an impact indicator for categorical data. For continuous variables, the mean difference (MD) with a 95% CI or standard mean difference (SMD) was used, depending on the measurement methods and units of the outcome indicators. The choice between a random-effects model and a fixed-effects model depended on the level of heterogeneity. When there was no significant heterogeneity (*I*^2^ < 50%), a fixed-effects model was employed; when there was considerable heterogeneity (*I*^2^ > 50%), a random-effects model was utilized. Sensitivity analysis was conducted to systematically exclude individual trials to investigate potential causes of heterogeneity. If clinical heterogeneity persisted after sensitivity analysis, a random-effects model was used to display the meta-analysis findings. Funnel plots were employed to assess publication bias.

### 2.5 Ethical approval

This systematic review and meta-analysis did not involve primary data collection from human participants or animals. However, the included studies were required to have obtained ethical approval from their respective institutional review boards or ethics committees. We ensured that all studies considered for inclusion adhered to ethical guidelines for human research as outlined by the Declaration of Helsinki. All studies included in this review have complied with ethical standards and have obtained appropriate informed consent from participants, where applicable. No personal or identifying information of individuals was included in the data extraction for this meta-analysis. The protocol for this systematic review and meta-analysis was prospectively registered with PROSPERO.

## 3 Results

### 3.1 Overview of the research

**Figure 1:**
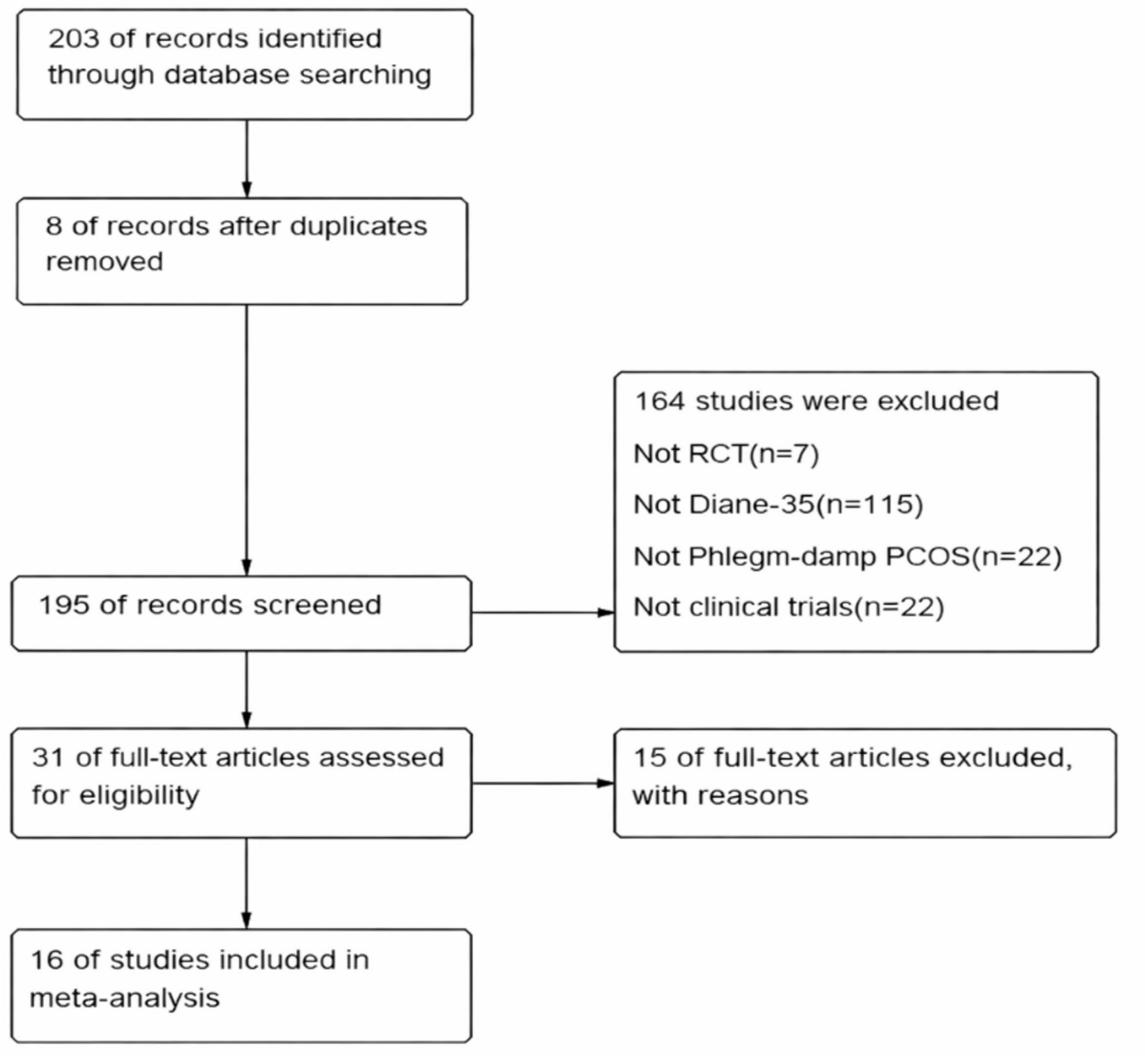
PRISMA flow diagram.

A total of 203 records were initially retrieved through database searching and other sources. After removing duplicate records, titles and abstracts were screened, leaving a subset of studies for full-text assessment. Following a detailed eligibility evaluation based on predefined inclusion and exclusion criteria, 16 studies were determined to meet all requirements and were therefore included in the final meta-analysis. The entire study selection process is summarized in Figure 1 (PRISMA flow diagram).

### 3.2 Study characteristics

A total of 16 studies were included in this meta-analysis, as detailed in Table 2. Sample sizes ranged from 22 to 60 participants, with balanced treatment and control groups across all studies. All included trials evaluated Cangfu Daotan-based prescriptions,including Cangfu Daotan Decoction, Modified Cangfu Daotan Decoction, and Cangfu Daotan Pill either alone or combined with Western medications.

**Table 2:** Characteristics of the studies.

No.	Trial	Sample size	Study design	Intervention	Control	Duration (menstrual cycles)	Diagnostic criteria	Outcome measures	Risk of bias
1	Xing Yan (2008) (23)	30/30	RCT	Modified Cangfu Daotan Decoction/Diane-35	Diane-35	3	Phlegm-Dampness Syndrome (TCM)	①③④⑥	High risk
2	Gao Jin (2013) (24)	30/30	RCT	Modified Cangfu Daotan Decoction/Diane-35	Diane-35	3	Phlegm-Dampness Syndrome (TCM)	③④⑤⑥	Moderate risk
3	Fu Yanhong (2017) (25)	22/20	RCT	Modified Cangfu Daotan Decoction/Diane-35	Diane-35	3	Phlegm-Dampness Syndrome (TCM)	①②③⑤	High risk
4	Xin Jun (2017) (26)	51/51	RCT	Cangfu Daotan Decoction/Diane-35	Diane-35	3	Phlegm-Dampness Syndrome (TCM)	①②③④⑤⑥	Low risk
5	Liu Hongxia (2018) (27)	45/45	RCT	Cangfu Daotan Decoction/Diane-35	Diane-35	3	Phlegm-Dampness Syndrome (TCM)	①③④⑥	Moderate risk
6	Sun Yuxiang (2018) (28)	40/40	RCT	Modified Cangfu Daotan Decoction/Diane-35	Diane-35	3	Phlegm-Dampness Syndrome (TCM)	①②③⑥	High risk
7	Xu Shiqian (2018) (29)	40/40	RCT	Modified Cangfu Daotan Decoction/Diane-35	Diane-35	3	Phlegm-Dampness Syndrome (TCM)	①②③④⑤⑥	Moderate risk
8	Liu Ying (2019) (30)	29/29	RCT	Cangfu Daotan Pill/Diane-35	Diane-35	3	Phlegm-Dampness Syndrome (TCM)	①②③④⑤	Low risk
9	Gao Xiujuan (2019) (31)	30/30	RCT	Modified Cangfu Daotan Decoction/Diane-35	Diane-35	2	Phlegm-Dampness Syndrome (TCM)	①②③⑤⑥	High risk
10	Gao Bingchun (2020) (32)	38/38	RCT	Cangfu Daotan Pill/Diane-35	Diane-35	3	Phlegm-Dampness Syndrome (TCM)	①②③⑥	Low risk
11	Zhao Ji (2021) (33)	54/54	RCT	Cangfu Daotan Decoction/Diane-35	Diane-35	4	Phlegm-Dampness Syndrome (TCM)	①②③	Moderate risk
12	Xing Xing-Ting (2023) (34)	30/30	RCT	Modified Cangfu Daotan Decoction + Diane-35	Diane-35	3	Phlegm-Dampness Syndrome (TCM)	①③④⑤⑥	High risk
13	Fu Yanhong (2024) (35)	36/41	RCT	Cangfu Daotan Decoction + Diane-35	Diane-35	3	Phlegm-Dampness Syndrome (TCM)	①②③⑤⑥	Moderate risk
14	Chen Li (2025) (36)	60/60	RCT	Cangfu Daotan Wan + Diane-35	Diane-35	3	Phlegm-Dampness Syndrome (TCM)	①②③④⑤⑥	Low risk
15	Wang Xiaotao (2025) (37)	38/38	RCT	Cangfu Daotan Decoction + Diane-35	Diane-35	4	Phlegm-Dampness Syndrome (TCM)	①②③⑥	Low risk
16	Dong Wenchao (2022) (38)	34/30	RCT	Modified Cangfu Daotan Decoction + Metformin + Diane-35	Metformin + Diane-35	3	Phlegm-Dampness Syndrome (TCM)	①②③④⑤⑥	Moderate risk

Cangfu Daotan Prescription including Cangfu Daotan Decoction (Decoction), cangfu Daotan pill (pill) and their modified type; T, treatment group; C, control group; CC, clomiphene citrate; ① LH ② FSH ③ T ④ LH/FSH ⑤ BMI ⑥ total effective rate.

Across the studies, the control groups primarily received Diane-35, whereas a subset used drospirenone–ethinylestradiol tablets (II) or metformin. Treatment durations varied between 2 and 4 menstrual cycles, with 3 cycles being the most commonly adopted regimen.

The outcome measures assessed across the included studies covered key endocrine and clinical parameters, including LH (①), FSH (②), testosterone (③), the LH/FSH ratio (④), BMI (⑤), and the total effective rate (⑥). Most studies reported testosterone, total effective rate, and LH, whereas fewer reported BMI and FSH. Overall, the included studies provided comprehensive data for evaluating the therapeutic effects of Cangfu Daotan-based interventions in PCOS management.

### 3.3 Methodological quality assessment

**Figure 2:**
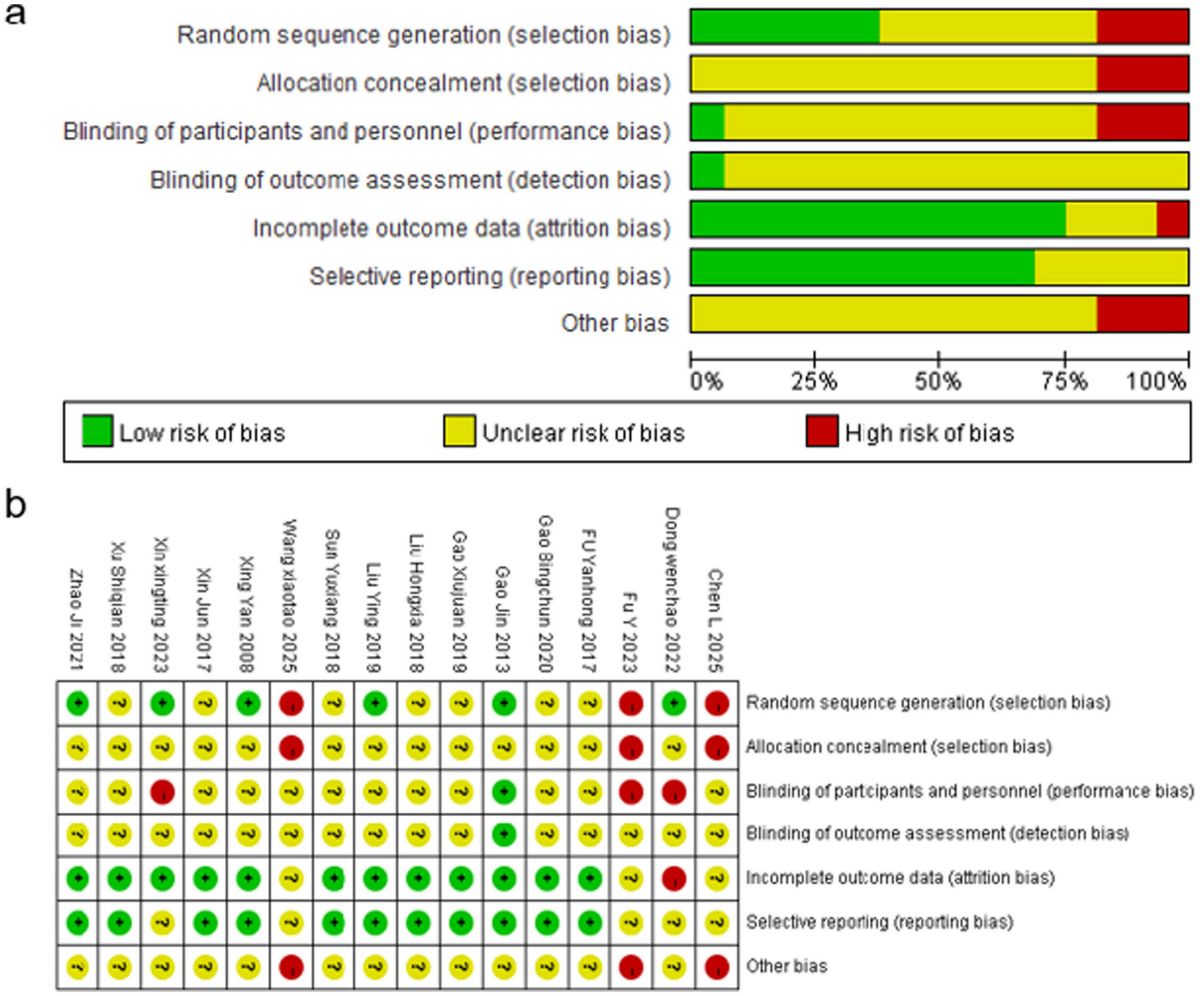
Risk of bias assessment for the included studies. **(a)** Summary of risk of bias based on the Cochrane Risk of Bias Tool, showing the proportion of studies rated as low risk (green), unclear risk (yellow), and high risk (red) across seven domains: random sequence generation, allocation concealment, blinding of participants and personnel, blinding of outcome assessment, incomplete outcome data, selective reporting, and other bias. **(b)** Risk of bias graph presenting judgments for each included study across the seven domains. Green circles indicate a low risk of bias, the yellow circles indicate unclear risk, and the red circles indicate high risk.

The methodological quality of the included studies was assessed via the Cochrane Risk of Bias Tool (Figures 2a,b). With respect to random sequence generation, 14 studies clearly described appropriate methods and were judged to have a *low risk of bias*, whereas two trials provided insufficient detail and were therefore rated as having an *unclear risk*. None of the studies reported allocation concealment procedures, leading to an *unclear risk of bias* for this domain across all 16 trials.

Blinding was generally not implemented. All studies lacked sufficient information on the blinding of participants and personnel, with 13 trials assessed as having *unclear risk* and three trials as *high risk* for performance bias. Similarly, none of the included studies reported blinding of outcome assessors, resulting in an *unclear risk* for detection bias in all studies.

Most studies demonstrated good data completeness, with 14 trials rated as having a *low risk* for incomplete outcome data. Selective reporting was judged as *low risk* in eight studies, whereas the remaining studies were categorized as *unclear*. For other potential sources of bias, four studies were rated as *high risk*, and the remaining 12 studies were judged to have an *unclear risk* due to insufficient methodological information.

### 3.4 Total effective rate

**Figure 3:**
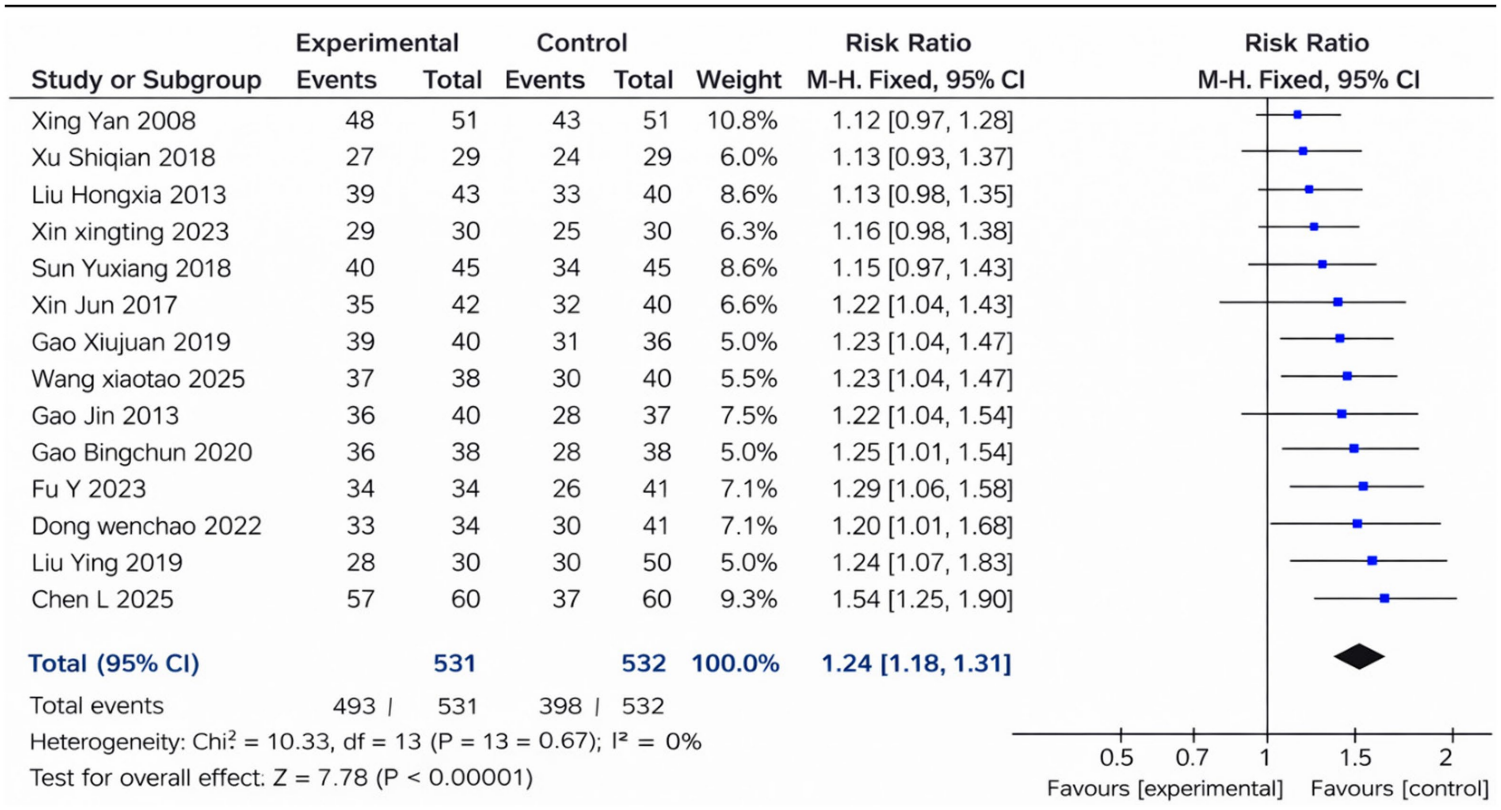
Forest plot of the total effective rate.

A total of 14 studies reporting the total effective rate were included in the pooled analysis (Figure 3). The combined results revealed that the Cangfu Daotan-based interventions significantly improved clinical efficacy compared with the control treatments (RR = 1.24, 95% CI 1.18–1.31, *p* < 0.00001). There was no heterogeneity among the studies (*I*^2^ = 0%, *p* = 0.67), and a fixed-effects model was applied. Overall, the pooled evidence indicates a consistently higher total effective rate in the treatment groups across trials.

### 3.5 Testosterone

**Figure 4:**
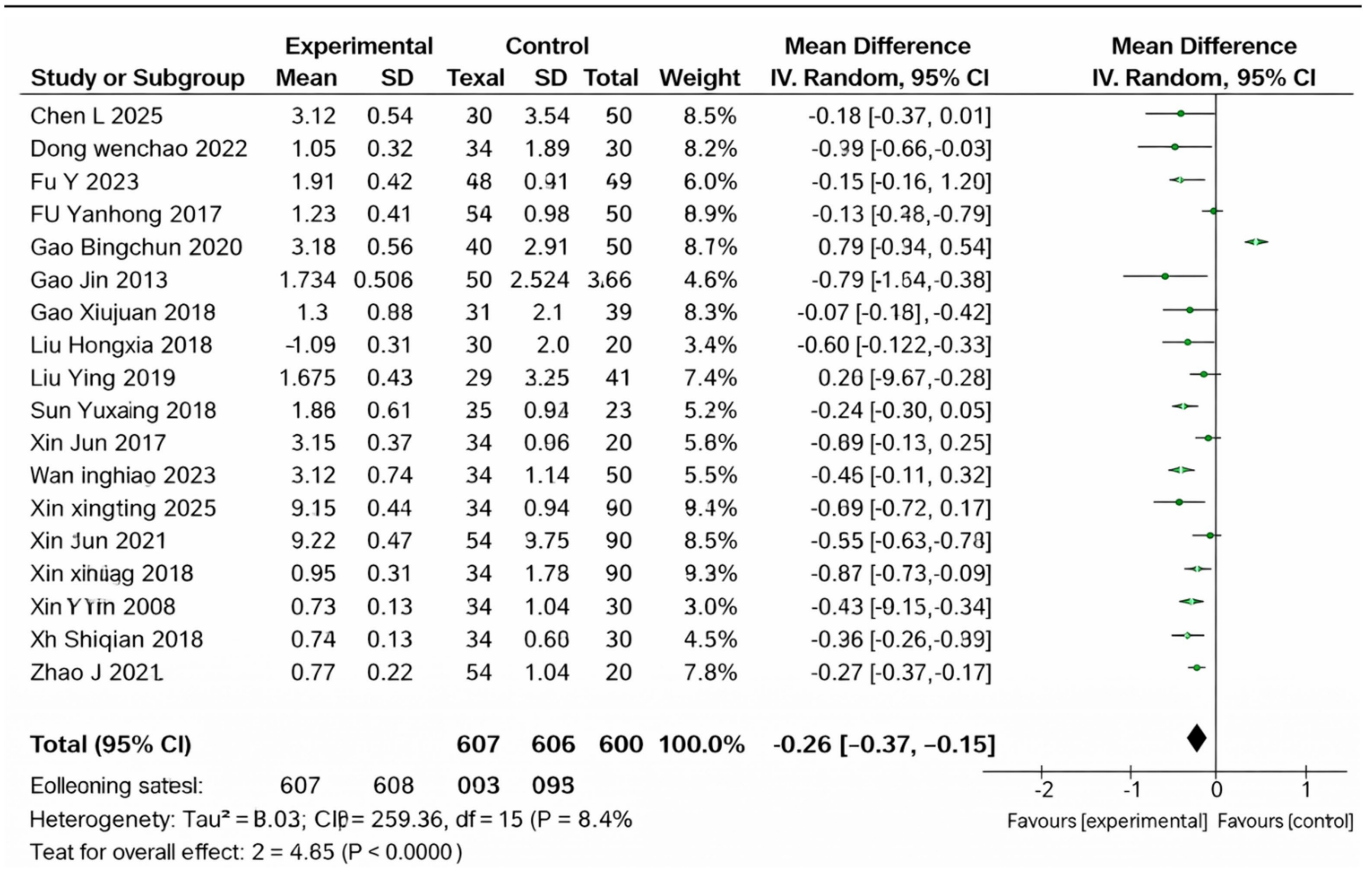
Forest plot of T.

A total of 16 studies reported testosterone levels and were included in the meta-analysis (Figure 4). The pooled results demonstrated that the Cangfu Daotan-based interventions significantly reduced serum testosterone compared with the control treatments (MD = −0.26, 95% CI –0.37 to −0.15, *p* < 0.00001). Although heterogeneity was substantial (*I*^2^ = 94%), all studies showed a consistent direction of effect favoring the treatment group. A random-effects model was therefore applied, and the overall evidence supported a clear reduction in testosterone levels among patients receiving Cangfu Daotan-based therapy.

### 3.6 Luteinizing hormone (LH)

**Figure 5:**
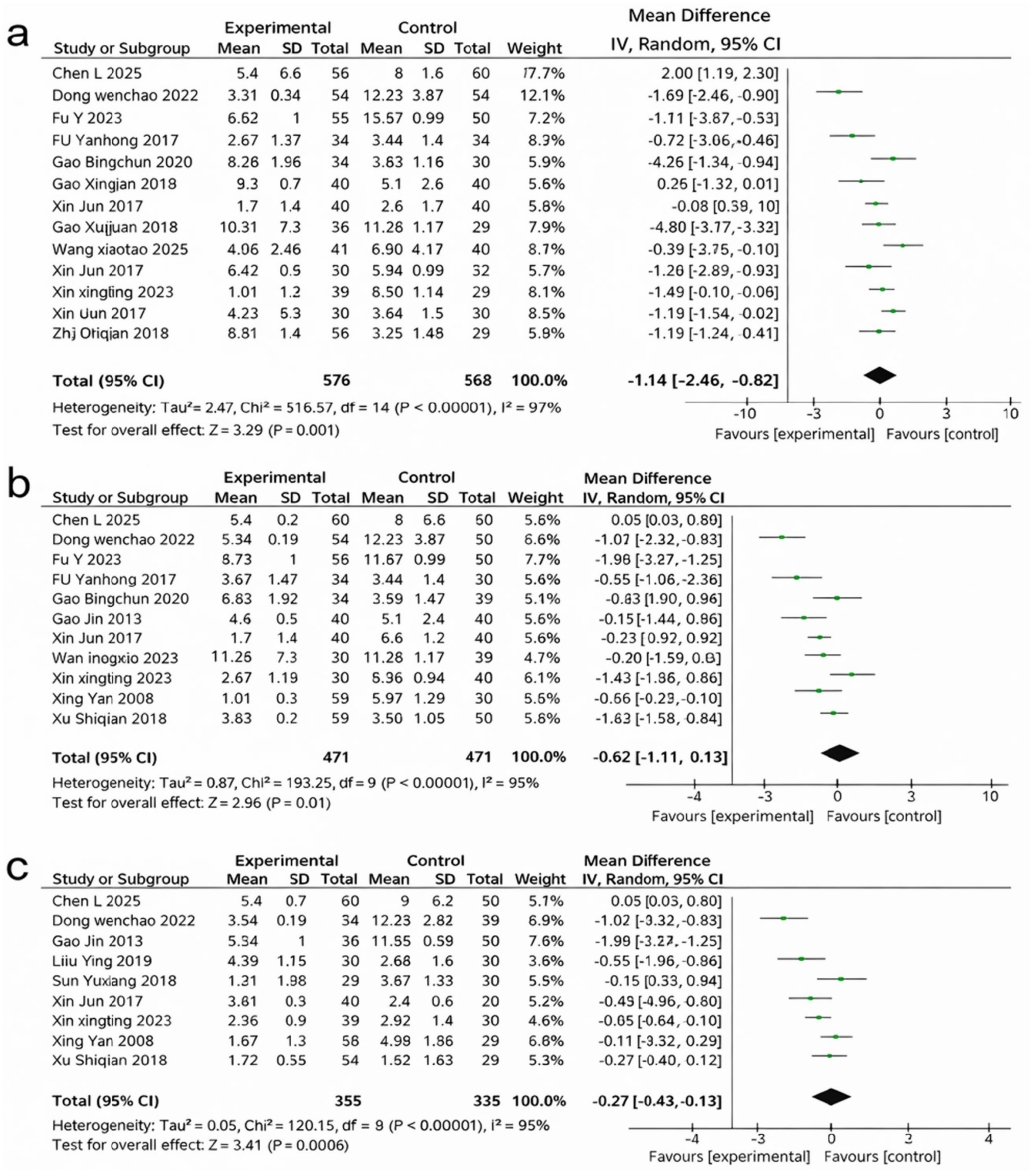
Forest plots of endocrine outcomes: **(a)** Forest plot of LH, **(b)** Forest plot of FSH, **(c)** Forest plot of LH/FSH.

A total of 16 studies reported LH levels and were included in the analysis (Figure 5a). The pooled results revealed a significant reduction in LH following Cangfu Daotan-based treatment compared with control interventions (MD = −1.64, 95% CI –2.46 to −0.82, *p* < 0.0001). Heterogeneity was considerable (*I*^2^ = 97%), and a random-effects model was applied. Despite the variability in effect sizes, all included trials demonstrated a consistent trend favoring LH reduction in the treatment group.

### 3.7 Follicle stimulating hormone (FSH)

Twelve studies reported FSH levels and were pooled in the meta-analysis (Figure 5b). The combined effect indicated a significant decrease in FSH in the treatment groups compared with the control groups (MD = −0.62, 95% CI –1.11 to −0.13, *p* = 0.01). Substantial heterogeneity was observed (*I*^2^ = 94%), and a random-effects model was used. Although the magnitude of reduction varied among studies, the overall direction of the effect consistently favored the treatment group.

### 3.8 Luteinizing hormone/follicle stimulating hormone (LH/FSH)

Nine studies provided data on the LH/FSH ratio (Figure 5c). The meta-analysis revealed a significant reduction in the LH/FSH ratio among participants receiving Cangfu Daotan-based therapy (MD = −0.27, 95% CI –0.43 to −0.12, *p* = 0.0006). Heterogeneity was high (*I*^2^ = 93%), and a random-effects model was applied. Across all included trials, the direction of effect consistently favored improvement in hormonal balance in the treatment group.

### 3.9 BMI

**Figure 6:**
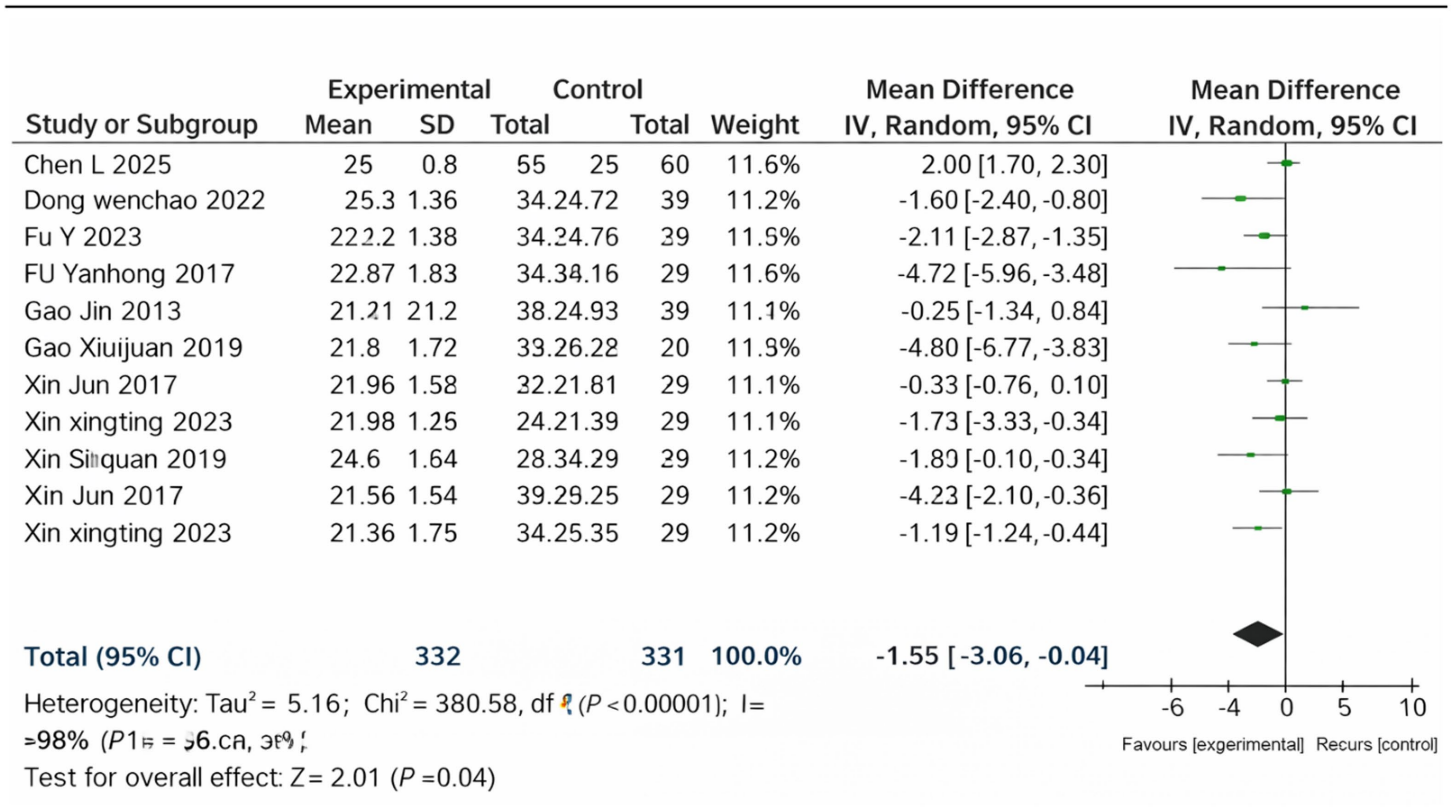
Forest plot of BMI.

Nine studies reported BMI outcomes and were included in the pooled analysis (Figure 6). The meta-analysis revealed that compared with the control interventions, the Cangfu Daotan-based interventions resulted in a significant reduction in BMI compared with control treatments (MD = −1.55, 95% CI –3.06 to −0.04, *p* = 0.04). Heterogeneity among studies was substantial (*I*^2^ = 98%), and a random-effects model was applied. Although the effect sizes varied, the overall direction consistently favored the treatment group, indicating a beneficial impact on weight-related outcomes.

### 3.10 Publication bias

**Figure 7:**
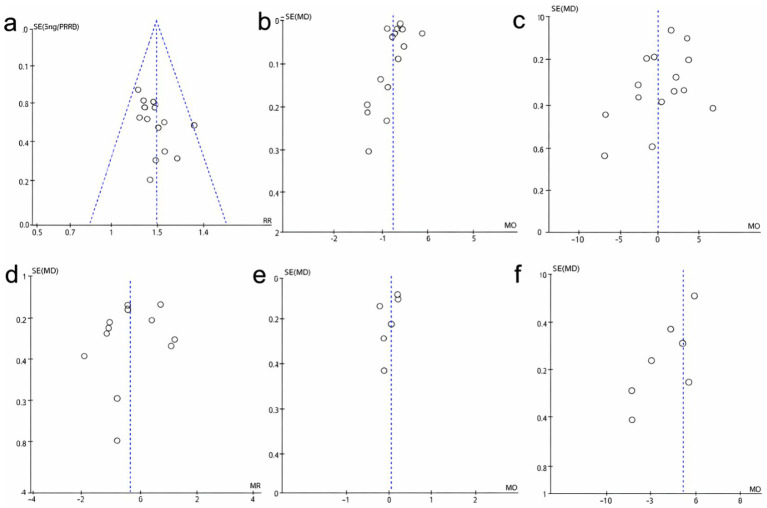
Funnel plots for publication bias. **(a)** Funnel plot of the total effective rate. **(b)** Funnel plot of T. **(c)** Funnel plot of LH. **(d)** Funnel plot of LH. **(e)** Funnel plot of LH/FSH. **(f)** Funnel plot of BMI.

Publication bias was evaluated via funnel plots for all outcomes (Figures 7a–f). The funnel plots for total effective rate, testosterone, LH, FSH, LH/FSH, and BMI all showed generally symmetrical distributions, indicating no clear evidence of publication bias among the included studies.

## 4 Discussion

### 4.1 Characteristics of phlegm-dampness PCOS

While TCM lacks a specific nomenclature for PCOS, it falls within the clinical spectrum of “amenorrhea” and “late menstruation” according to its clinical characteristics. Among the various types of PCOS in TCM, nearly 43% are identified as phlegm-dampness PCOS. In addition to typical clinical manifestations such as a white and greasy tongue coating, a plump abdomen, and phlegm, phlegm-dampness in patients with PCOS presents distinct characteristics in terms of clinical detection indices. According to Fang et al. (10), PCOS patients with phlegm dampness syndrome presented higher testosterone (T) levels compared to those without phlegm-dampness syndrome. Lipin et al. (11) supports these findings, indicating no significant difference in LH and LH/FSH levels between the phlegm-dampness PCOS patients and other PCOS patients. Logistic binomial regression analysis of phlegm-dampness PCOS patients revealed that factors such as weight, and BMI, increase the risk of phlegm dampness syndrome in PCOS patients (12).

The diagnostic criteria for phlegm-dampness syndrome in individuals with polycystic ovary syndrome (PCOS) varied across the included studies. While all the studies adhered to Traditional Chinese Medicine (TCM) pattern identification for phlegm-dampness syndrome, the specific criteria used to define the syndrome were not always consistent. The studies followed different TCM textbooks, clinical guidelines, or consensus statements, which resulted in variations in the exact symptom criteria (e.g., number of symptoms required for diagnosis) and the interpretation of tongue and pulse signs. For example, some studies applied national TCM diagnostic standards, while others referenced more traditional or recently updated TCM resources.

Despite these variations, common clinical features such as overweight, abdominal fullness, and sticky leukorrhea were consistently reported as key diagnostic indicators. To ensure transparency, we have provided a summary of the specific TCM standards and diagnostic criteria used in each study in Table 1, which outlines the differences and similarities in diagnostic approaches. This clarification ensures that readers are aware of the potential impact of these variations on the clinical outcomes and overall findings of our review.

### 4.2 Effects Cangfu Daotan decoction

PCOS patients typically require long-term treatment, and TCM offers unique advantages, including greater patient acceptance (especially for Chinese individuals), fewer side effects, and lower costs. TCM’s distinctive feature of “differential diagnosis and treatment” emphasizes treating the syndrome rather than specific diseases. This approach is based on the principle that the same syndrome reflects the same pathomechanism, and that treatment should align with individual patient characteristics.

Cangfu Daotan decoction, a clinical TCM decoction, is primarily used to address excess phlegm, Qi deficiency, menorrhea, leucorrhea, and obesity. The composition of Cangfu Daotan decoction includes Atractylodes, Glycyrrhiza, Poria cocos and Pinellia ternate, which invigorate the spleen and dampness. Fructus aurantii can break Qi and eliminate accumulation. Dried tangerine peel can reduce Qi and phlegm. Bile arisaema can also reduce phlegm, regulate the stomach and stop vomiting. Ginger juice, relieves the toxicity of *Pinellia ternata* and Bile arisaema, and reduces adverse reactions and Nutgrass galingale rhizome, disperses stagnated liver (13).

According to modern pharmacological research, Cangfu Daotan decoction clearly regulates reproductive endocrine hormone levels and insulin resistance of phlegm-dampness-related PCOS (14). Although its molecular mechanism is not fully elucidated, Cangfu Daotan decoction has been found to up-regulate the expression of Organic Anion Transporting Polypeptide 3A1 (OATP3A1) in the spleen, liver, kidney and other tissues. This enhances the ability of these organs to transport phlegm dampness, alleviating the symptoms of phlegm dampness blockade in PCOS (15). Li et al. (16) discovered that Cangfu Daotan decoction reduces PKP3 expression via improving the methylation of the PKP3 promoter, which causes an increase in cells in the S and G2/M phases of the cell cycle, stops the apoptotic process, and promotes the proliferation of ovarian granulosa cells. Chen et al. (17) reported that FOXK1 has an inhibitory effect on DNA synthesis in the ovarian granulosa cells of PCOS rats, whereas Cangfu Daotan decoction can reverse the autophagy effect of FOXK1 on granulosa cells in PCOS rats. This research indicated that FOXK1 may be a new target of Cangfu Daotan Formula in the treatment of PCOS. In addition, in the treatment of PCOS-related diseases, Hao (18) reported that Cangfu Daotan decoction may contribute to improvements in endocrine- and metabolism-related profiles in individuals with PCOS, including the modulation of reproductive-hormone levels and insulin resistance. While the recent meta-analysis offers a comprehensive overview (19), our study provides additional value by including more recent RCTs and offering a detailed subgroup analysis focusing on specific clinical outcomes. Our study also considers a broader range of clinical parameters, providing a more nuanced understanding of the effects of Cangfu Daotan decoction on different patient populations. Furthermore, we address some limitations in the recent meta-analysis, such as the lack of detailed subgroup analyses and the inclusion of the latest data.

A systematic review confirmed that Cangfu Daotan Decoction exerts a certain effect on the adjuvant treatment of PCOS, but clearly indicates that its efficacy in improving FSH levels is limited this conclusion has long been the mainstream perception in the academic community regarding the effect of Cangfu Daotan Decoction in treating PCOS.

However, this study provides a conclusion contrary to previous research for the first time: Cangfu Daotan Decoction can effectively improve follicle-stimulating hormone levels. Our pooled analysis revealed that FSH was modestly lower in the combination group; however, the heterogeneity was substantial and the clinical interpretation of FSH changes in PCOS should be cautious. Differences from previous reviews may be related to study selection, population characteristics, and pattern-identification criteria. As the core pathological basis of phlegm-dampness type PCOS, the accumulation of phlegm-dampness in the body can directly induce insulin resistance; in addition, it can interfere with the normal regulatory mechanism of the hypothalamic–pituitary axis on follicle-stimulating hormone, leading to abnormal FSH levels. Cangfu Daotan Decoction can significantly improve patients’ insulin sensitivity, thereby indirectly regulating the secretion rhythm of pituitary follicle-stimulating hormone and targeting abnormal FSH levels in patients with phlegm-dampness type PCOS. While this meta-analysis and systematic review provide valuable insights into the efficacy of Cangfu Daotan decoction combined with Diane-35 in treating phlegm-dampness-related PCOS, several limitations exist. First, many of the included studies had an unclear risk of bias due to insufficient details on random sequence generation, allocation concealment, and blinding. This may affect the reliability of the results. Second, significant heterogeneity was observed in some outcome measures, such as BMI. Although sensitivity analyses were performed, the sources of heterogeneity could not be completely identified. A common outcome reported in the included studies was the “total effective rate,” which reflects the proportion of patients classified as “recovered,” “markedly improved,” or “improved.” While this outcome is frequently used in traditional Chinese medicine (TCM) clinical trials, it has several limitations. The “total effective rate” is subjective and its definition is not standardized across studies, which may lead to inconsistent interpretations and reporting. Given the variability in how this outcome is defined, the reliance on it as a primary measure in this review could introduce bias and limit the comparability of results across studies. To address this concern, we used more objective outcomes, such as serum testosterone levels, LH/FSH ratios, and BMI, wherever available, to supplement the analysis. These more standardized measures provided additional insights into the effects of the interventions on the clinical outcomes of PCOS. Future research should aim to use more consistent and validated measures to assess treatment efficacy, reducing the reliance on subjective outcomes like the “total effective rate.” Additionally, adverse events (AEs) are an important aspect of evaluating the safety of any medical intervention. However, many of the included studies did not systematically report adverse events, or the reporting was inconsistent across studies. When adverse events were reported, they were often presented in a non-standardized format, making it difficult to compare across studies. In those studies that did report AEs, the most commonly mentioned adverse events included mild gastrointestinal discomfort, fatigue, and dizziness. Serious adverse events were rare and were not consistently reported. To address this, we systematically summarized the adverse events across the included studies and found that the overall incidence of AEs was relatively low, with mild symptoms being the most frequent. However, future studies should place greater emphasis on standardizing the reporting of adverse events, providing clear definitions and consistent reporting formats, to ensure that the safety profile of interventions like Cangfu Daotan decoction combined with Diane-35 can be adequately assessed.

Finally, while some studies have suggested potential mechanisms of action for Cangfu Daotan decoction, the exact molecular mechanisms remain unclear and warrant further investigation.

The majority of the included studies in this systematic review presented a high risk of bias, particularly due to unclear randomization methods, absent blinding, and the lack of allocation concealment. These methodological limitations could introduce bias in both the selection of participants and the assessment of outcomes. As a result, the findings from these studies should be interpreted with caution. Although we performed a risk of bias assessment using the Cochrane Risk of Bias Tool, the high number of studies with unclear randomization and blinding could affect the internal validity of the results. To assess the potential impact of this bias, we conducted sensitivity analyses to examine whether the inclusion of studies with high risk of bias significantly influenced the overall results. However, despite these efforts, the lack of robust methodological quality in the included studies remains a limitation of our review. Therefore, future research with improved methodological rigor, particularly clearer randomization, blinding, and allocation concealment, is essential to confirm the findings presented in this review.

### 4.3 High heterogeneity and subgroup analyses

A significant level of heterogeneity (*I*^2^ > 90%) was observed in some of the analyses, particularly in relation to the effects of the interventions on clinical outcomes. This high heterogeneity may be attributed to several factors, including variations in intervention type, treatment duration, and the use of co-medications across the included studies. For example, some studies used different formulations of Cangfu Daotan, while others varied in the duration of the intervention, which could lead to differences in treatment outcomes. Additionally, some studies combined Cangfu Daotan with other medications such as Diane-35, metformin, or other hormonal therapies, further complicating the comparison of results.

To address this, we conducted subgroup analyses based on intervention type (e.g., Cangfu Daotan decoction vs. modified formulations), treatment duration, and the use of co-medications. These subgroup analyses provided further insight into the sources of heterogeneity and helped identify the most consistent effects of Cangfu Daotan for different patient populations and treatment regimens. However, substantial heterogeneity remained, indicating that factors beyond the intervention type and duration may also play a role in the variability of outcomes.

Given the high heterogeneity, we suggest that future studies should aim for more standardized interventions with clear reporting on treatment duration and co-medications to reduce variability and allow for more consistent meta-analysis. Furthermore, conducting more rigorous subgroup analyses would be valuable in understanding how these variations influence treatment efficacy.

While this meta-analysis provides valuable insights into the potential benefits of Cangfu Daotan decoction combined with Diane-35 in treating phlegm-dampness PCOS, the findings must be interpreted with caution. Many of the included studies had unclear or high risks of bias, particularly in randomization, blinding, and allocation concealment. These methodological limitations could affect the internal validity of the studies and the reliability of the overall results. Additionally, significant heterogeneity was observed across the studies, which could be attributed to variations in intervention types, treatment durations, and the use of co-medications. Despite conducting subgroup and sensitivity analyses, substantial heterogeneity remains, and the sources of this variability are not fully understood. Furthermore, the outcome measures employed were not always consistent or standardized, which may affect the comparability of results across studies.

Given these limitations, it is important to interpret the findings of this review as preliminary and exploratory. More rigorous, well-designed clinical trials with clearer reporting and improved methodological quality are needed to confirm the efficacy of Cangfu Daotan decoction in treating phlegm-dampness PCOS. Future research should also aim to establish more standardized diagnostic criteria and outcome measures to reduce heterogeneity and increase the reliability of the evidence.

## 5 Conclusion

This systematic review and meta-analysis suggest that Cangfu Daotan decoction, when combined with Diane-35, may have beneficial effects on patients with phlegm-dampness type PCOS, particularly in improving certain clinical parameters such as hormone levels and insulin sensitivity. However, the low quality of the included studies, with many presenting a high risk of bias, limits the strength of the evidence and the generalizability of the findings. The conclusions drawn from this analysis should therefore be interpreted with caution, and the results should be viewed as preliminary, warranting further confirmation through well-conducted, high-quality clinical trials. While some mechanistic explanations, such as the regulation of insulin resistance and the modulation of hormone levels, were proposed, these are based on theoretical and preclinical findings rather than clinically validated data. More rigorous clinical studies are needed to validate these mechanisms and to assess their true impact on the treatment of PCOS. Future research should focus on improving the methodological quality of studies, using standardized measures for both efficacy and safety, and further exploring the clinical relevance of the proposed mechanisms of action. Randomized controlled trials with larger sample sizes and more consistent reporting will be essential to establish the therapeutic value of Cangfu Daotan decoction for phlegm-dampness PCOS.

## Data Availability

The raw data supporting the conclusions of this article will be made available by the authors, without undue reservation.
